# Regulatory Multitasking of Tolerogenic Dendritic Cells – Lessons Taken from Vitamin D3-Treated Tolerogenic Dendritic Cells

**DOI:** 10.3389/fimmu.2013.00113

**Published:** 2013-05-14

**Authors:** Tatjana Nikolic, Bart O. Roep

**Affiliations:** ^1^Department of Immunohematology and Blood Transfusion, Leiden University Medical CenterLeiden, Netherlands

**Keywords:** tolerogenic dendritic cells, vitamin D, regulatory T cells, autoimmune diseases, costimulatory molecules

## Abstract

Tolerogenic dendritic cells (DCs) work through silencing of differentiated antigen-specific T cells, activation and expansion of naturally occurring T regulatory cells (Tregs), transfer of regulatory properties to T cells, and the differentiation of naïve T cells into Tregs. Due to an operational definition based on T cell activation assays, the identity of tolerogenic DCs has been a matter of debate and it need not represent a specialized DC subset. Human tolerogenic DCs generated *in vitro* using inhibitory cytokines, growth factors, natural immunomodulators, or genetic manipulation have been effective and several of these tolerogenic DCs are currently being tested for clinical use. *Ex vivo* generated tolerogenic DCs reduce activation of naïve T cells using various means, promote a variety of regulatory T cells and most importantly, frequently show stable inhibitory phenotypes upon repetitive maturation with inflammatory factors. Yet, tolerogenic DCs differ with respect to the phenotype or the number of regulatory mechanisms they employ to modulate the immune system. In our experience, tolerogenic DCs generated using the biologically active form of vitamin D (VD3-DCs), alone, or combined with dexamethasone are proficient in their immunoregulatory functions. These tolerogenic DCs show a stable maturation-resistant semi-mature phenotype with low expression of activating co-stimulatory molecules, no production of the IL-12 family of cytokines and high expression of inhibitory molecules and IL-10. VD3-DCs induce increased apoptosis of effector T cells and induce antigen-specific regulatory T cells, which work through linked suppression ensuring infectious tolerance. Lessons learned on VD3-DCs help understanding the contribution of different pattern-recognition receptors (PRRs) and secondary signals to the tolerogenic function and how a cross-talk between DCs and T cells translates into immune regulation.

## Introduction

Dendritic cells (DCs) comprise a network of professional antigen-presenting cells (APC) throughout the body that orchestrate responses to foreign antigens, while keeping in check autoreactive T cells (Steinman et al., 2003a). DCs may initiate (inflammatory DCs) or inhibit (tolerogenic DCs) the antigen-specific response. Apart from preventing pro-inflammatory responses to antigens, tolerance comprises active inhibition of existing effector immune responses against a particular antigen. Depending on the origin of this antigen, we may differentiate tolerance for self-antigens, for antigens that originate in the environment or for alloantigens after transplantation. Breaking tolerance may lead to pathology in all cases: reaction of the adaptive immunity against alloantigen may cause graft rejection; intolerance to foreign antigen predisposes to allergy while immune responses against self-antigen may lead to development of autoimmune disease.

There are different mechanisms by which immune tolerance can be achieved: for instance, removal of pathogenic T cells, inducing, or restoring a balance between pro- and anti-inflammatory immunity or by action of regulatory T cells (Treg). A superior ability of DCs to interact with naïve and experienced T cells and contribute to all mechanisms of immune tolerance makes these attractive candidates for tolerance induction strategies. Tolerogenic DCs prevent the destructive action of self-reactive T cells that had escaped the selection in the thymus, by mechanisms of peripheral tolerance (Steinman et al., 2003a). Stimulation of Th1 and/or Th2-type of the immune response, which sometimes balances the destructive immunity, is also critically dependent on action of DCs (Singh et al., 1999; Kidd, 2003; Amsen et al., 2004). Finally, DCs and Treg are engaged in active dialog and critically depend on each other’s function. Induction and maintenance of Treg depends on DCs while Treg employ their regulatory action through direct interaction with DCs, in addition to affecting effector T cells (Vendetti et al., 2000; Salcido-Ochoa and Lechler, 2002; Pasare and Medzhitov, 2003; Steinman et al., 2003b).

Tolerogenic mechanisms essentially employ the antigen-presenting function of DCs, in which co-stimulatory molecules play an important role. Activation of T cells requires that at least two signals are transferred by the APC: (Steinman et al., 2003a) antigen in the form of peptide bound to MHC molecules (Amsen et al., 2004), co-stimulation, especially provided by molecules of the B7 and TNF-receptor families. In addition, cellular adhesion molecules and cytokines support activation of T cells that have made a productive interaction with antigen-presenting- and co-stimulatory molecules. For instance, inflammatory DCs produce and present IL-2 to naïve T cells to promote their activation (Granucci et al., 2002; Wuest et al., 2011), cytokines of the IL-12 family and IL-4 skewing T cells into a T helper-1 (Th1) or T helper-2 (Th2) polarized phenotype (Kalinski et al., 1999), and DCs secrete IL-10, TGFβ, IL-35, or TNF, which promote generation of regulatory T cells (Kushwah and Hu, 2011). Finally, stimulated T cells may be endowed with tissue-specific homing capacities as a “fourth” signal delivered by APC to T cells (Campbell and Butcher, 2002; Villablanca et al., 2008). The quantity and quality of the distinct signals delivered by APC determine the outcome of the ensuing T cell response and the ability of DCs to activate a strong effector cell response has been vastly appreciated. However, DCs are also crucial in downregulation and tolerization of the adaptive immune system and apart from being able to sense various danger signals in inflammation, DCs integrate tolerogenic signals originating from peripheral or lymphoid tissues, supporting the maintenance of immune homeostasis and avoidance of T cell autoreactivities (Steinman et al., 2003a; Yogev et al., 2012). The primary function of DCs thus might be induction and maintenance of immune tolerance rather than activation of effector responses.

Approaches to harness the immunoregulatory function of DCs encompass the search for tolerogenic DCs *in vivo* and the *in vitro* manipulation of monocyte-derived DCs to confer and secure their tolerogenic function. The emerging concept implies that *in vivo* tolerogenic DCs might not represent a specialized DC subset but might embody a particular state of DC differentiation influenced by a collection of tissue and environmental factors, making it difficult to manipulate these cells in real time. The interplay between resident DCs and naturally occurring Tregs (tTreg) from the thymus likely reinforces the default tolerogenic state of DCs. *In vitro* manipulation of monocyte-derived DCs has proven useful and modulated DCs are being currently tested for clinical use. In this review, we summarize our lessons learned using Vitamin D3-treated DCs on the role of secondary signals in the generation of tolerogenic DCs and how these may induce and maintain peripheral tolerance.

## Heterogeneity of Tolerance-inducing Dendritic Cells: Do Tolerogenic Dendritic Cells Represent a Distinct Subset?

The highly increased awareness of the role of DCs in steering the immune system and their possible clinical applications has resulted in a wealth of information about the phenotypic heterogeneity of DCs. However, unequivocal interpretation of these findings in developmental and functional terms has proven to be difficult, especially because of the high turn-over of DCs, their significant mobility between peripheral and immune organs, the phenotypic volatility of these cells in response to environmental conditions, and the differences between strategies to endorse tolerogenic capacity. The application of phenotypic labeling to define DCs created confusion since many “new” DC types in different organs were defined, without a clear view on the possible developmental relationships between these and already known DC types. To illustrate this: in human thymus, three distinct DC populations have been found up to five populations in lymph nodes and tonsils and at least three populations in blood (Lewis and Reizis, 2012). The situation is similarly complex in mice, since up to six different DC types were found in mouse lymph nodes and spleen (Dominguez and Ardavin, 2010).

Understanding the *in vivo* function of these different DCs and establishing the identity of tolerogenic DCs has consequently taken a while. In general, two schools of thinking have arisen; one that proposes tolerogenicity to be a functional property of DC at a particular (immature) stage in development and another that hypothesizes that a specific tolerogenic DC lineage exists. In favor of the first proposal, immature DC induce anergic T cells, T cells with regulatory properties as well as T cells that secrete immunomodulatory cytokines. However, the immature phenotype of DCs is replaced by mature/inflammatory upon triggering in the periphery by danger/inflammatory signals and migration to the lymph nodes, making the tolerogeneicity of immature DCs a transient phase that can hardly be controlled for therapeutic purposes. Establishment of DCs with a “semi-mature” phenotype, representing cells that have been “matured” in the absence of inflammation, has enabled definition of functional regulatory DCs with a stable phenotype (Lutz and Schuler, 2002). The phenotype of these DCs may vary depending on the “tolerizing” signals they receive but in general, semi-mature DCs are able to present antigens like mature DCs but lack high level pro-inflammatory cytokine production and their repetitive injection prevents autoimmunity in experimental models.

The hypothesis that a specific lineage of DC induces tolerance has been widely accepted as well (Coquerelle and Moser, 2010). Studies demonstrating two types of DCs in the peripheral blood, so called myeloid or conventional (cDCs) and plasmacytoid DCs (pDCs), which had different origins and functions, supported the notion that inflammatory and tolerogenic DCs are unrelated cells with separate precursors. In mice, CD8 – cDCs and tissue-derived cDCs are predominantly immunogenic. Most notably implicated in tolerance induction are CD8+ cDCs (Yamazaki et al., 2008) and pDCs (Nikolic et al., 2009), so these were logical candidates for a special tolerogenic DC lineage in mice. However, CD8+ cDCs are not only tolerogenic but also cross-present antigens and prime CD8 T cells (Crozat et al., 2011). Similarly, the tolerogenic potential of pDCs has been demonstrated in allergic inflammation *in vivo* (Kool et al., 2009), but pDCs can also stimulate Th1 or Th2 immunity and CD8 T cell responses. This indicates that both CD8+ cDCs and pDCs can perform distinct functions depending on the sum of factors that had influenced their maturation and activation (Swiecki and Colonna, 2010; Takagi et al., 2011). Together with the identification of a common precursor for cDC and pDC in mice (Auffray et al., 2009), the notion of a separate tolerogenic DC lineage became less plausible.

**Figure 1:**
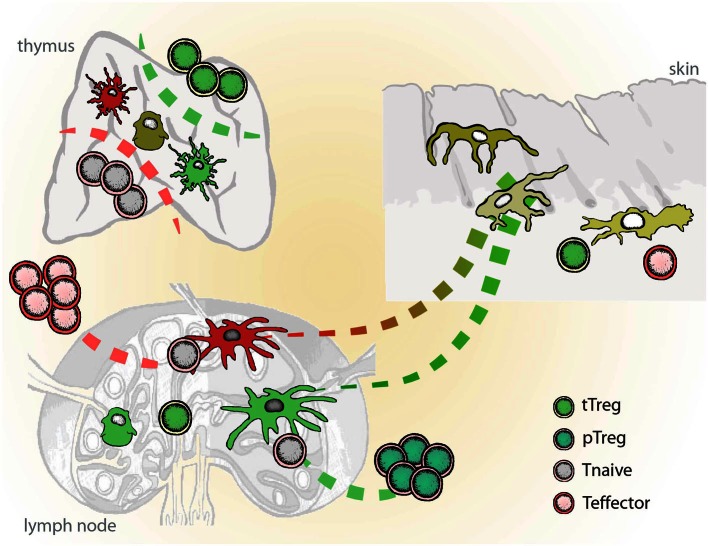
**Schematic representation of inflammatory vs. tolerogenic DCs *in vivo***. Inflammatory DCs (red) differentiate from the immature tissue-resident DCs and migrate to the lymph nodes, where they initiate immune responses and instruct generation of the effector T cells (Teff). Semi-mature DCs (green), which develop from the tissue-resident DCs in the absence of danger and inflammatory signals, induce tissue-specific adaptive regulatory T cells (pTreg). Dendritic cells in thymus play a role in the positive and negative selection of naïve lymphocytes (Tnaive) and the induction of naturally occurring regulatory T cells (tTreg). In mice, DCs seem to play dispensable role in the thymus.

Since both cDC and pDC as found in blood, bone marrow, or peripheral organs represent the immature stage of the cell, the notion is that “immature” or migratory DCs (plasmacytoid or conventional) are able to down-regulate immunity until they encounter an activation stimulus and become immunostimulatory APC. This again strengthens the view that all DC are in principle tolerogenic in steady state, unless triggered by a “danger signal” in the inflammatory context. Therefore, a unifying platform allowing both scenarios under particular conditions would be that by conditioning during differentiation determines the functional plasticity of DCs and their diversity in origin/phenotype has evolved to enable the immune system to “sense” various danger signals with different intensity leading to different functional outcomes depending on the context (Figure 1).

## Harnessing Tolerogenic DCs for Therapeutic Purposes

Dendritic cells that mediate tolerance induction *in vivo* exist without doubt. However, it is still debatable whether a particular function demonstrated *in vitro* may also be exerted *in vivo*. Immune tolerance can be achieved through different mechanisms and the experimental data from an *in vivo* system do not discern whether a particular DC subtype employs one or more mechanisms, or what other cells in the system might contribute. For example, thymic DCs have been associated with selection of developing T cells, but a recently established mouse model lacking the majority of DCs (DC-less mice) suggested that DCs are dispensable in thymic negative selection (Yogev et al., 2012). Yet, DCs in the periphery remain essential in induction and maintenance of peripheral tolerance, since DC-less mice are able to mount an immune response but fail to regulate it and develop enhanced EAE and progressive myeloproliferative disorders, resulting in spontaneous fatal autoimmunity (Birnberg et al., 2008; Ohnmacht et al., 2009; Yogev et al., 2012). The expression of the MOG auto antigen in steady state DCs in the brain induced strong tolerance, correlating with the increased expression of PD-1 on adaptive regulatory T cells (Yogev et al., 2012). Taken together, these studies show: (i) that autoimmunity may be activated by other cells than DCs; (ii) that DCs are essential for the maintenance of tolerance; (iii) that DCs may act by enhancing adaptive regulatory T cells.

Human peripheral blood pDCs and a subtype of human blood DCs that expresses CD141 (CD141+ DCs) are believed to mediate tolerance *in vivo*. Although, there is some phenotypic and functional overlap between human and murine DCs, there are also important differences that prevent straightforward translation of findings between species. For example, while both human and mouse pDCs selectively express TLR7 and TLR9, securing the ability to produce IFN-I in response to TLR7/9 ligation, human pDCs do not produce IL-12p40, which may contribute to their tolerogenic capacities. Both human and murine pDCs express CXCR3 and CXCR4, but human pDCs lack CCR9 expression, which could be related to different maturation state (Drakes et al., 2009). CCR9 is expressed by human CD141+ DCs, which are considered to be a homolog of mouse CD8+ DCs (Jongbloed et al., 2010; Poulin et al., 2010). Yet, human CD141+ DCs do not express the same TLR receptors as the mouse counterpart, thus have different sensor functions (Kaisho, 2012). Skin resident CD141+ DCs are the major producer of IL-10, they induce T cell anergy and Tregs that suppress skin inflammation. Interestingly, treatment with vitamin D3 induces skin CD141+ DC-like phenotype on human CD141− DCs (Chu et al., 2012).

Manipulation of mouse DCs *in vivo* has shown therapeutic potential to control immune response. Selective depletion of mDCs sparing pDCs strongly attenuated autoimmune response and reduced diabetes incidence in NOD mice (Nikolic et al., 2005), and specific selective expansion of pDCs *in vivo* reduce strong allergic responses (Kool et al., 2009). Depletion of a subtype of dermal DCs prevents differentiation of effector Th cells and conferred resistance to EAE (King et al., 2010), and elimination of Langerhans cells in the skin decreased the low-dose contact hypersensitivity (Kel et al., 2010). A fine tuning between CD8+ and CD8− cDCs that differentially stimulate Th1 vs. Th2 responses can regulate immunity, but the potential of DCs to stimulate a particular type of helper T cells depends on the type of the stimulatory signal they encounter. Immunoregulatory function of tolerogenic DCs *in vivo* is usually associated with their incompletely differentiated state. It would be important to learn how to prevent DCs *in vivo* from acquiring immunizing abilities to avoid lack of control and offer safe therapeutic application in humans.

## *In vitro* Generated Tolerogenic DCs

Monocytes may be manipulated *in vitro* to generate DCs with desired function and this has been investigated as immunotherapy to a great extent (Palucka et al., 2003; Figdor et al., 2004; Germain, 2010; Chia et al., 2012; Hong et al., 2012; Palucka and Banchereau, 2012). Development of DCs *in vitro* under influence of GM-CSF and IL-4, reconstructs the sequence of events established for peripheral tissue DCs although a direct approximation of the *in vitro* generated DCs to an *in vivo* existing subset is not possible. Tissue-resident DCs, like immature DCs in culture, are characterized by high endocytic capacity and low surface expression of major histocompatibility complex (MHC) and co-stimulatory molecules. Danger signals or inflammation encountered in the periphery triggers maturation of DCs, enabling these to migrate to the lymph nodes and on their way acquire all necessary tools to initiate an adequate response to phagocytosed antigens. The maturation *in vitro* is induced by stimulating DCs through pattern-recognition receptors (PRRs), CD40, or by inflammatory cytokines.

Different approaches have been tested to induce tolerogenic DCs *in vitro* and the features DCs have to fulfill are related to the semi-mature phenotype, resistance to maturation, anti-inflammatory cytokine profiles, and induction of specific T-cell profiles. A tolerogenic state of DCs can be induced using several pharmacological agents such as rapamycine, dexamethasone, Vitamin A, or Vitamin D, cytokines such as IL-10 or different growth factors such as G-CSF, VEGF, VIP, and many others (Rutella et al., 2006). Alternative approach has been tested as well using antisense nucleotides to enforce stable immature phenotype of DCs (Machen et al., 2004). These different tolerogenic DCs may share certain features, such as semi-mature phenotype or the ability to suppress allo-reactive response in the mixed leukocyte reaction (MLR), yet many have specific features not shared by others. An overview of phenotype and functional properties of different *ex vivo* generated DCs is given in Table 1.

**Table 1:** **Protocols reported to date that generate tolerogenic DCs from human monocytes**.

Modulator	Treatment start	HLA-DR and co-stimulatory molecules	Inhibitory molecules	Cytokine production	Inhibition of T cells*	Induction of Tregs	Additional characteristics	Reference
IL-10	Monocytes	HLA-DR low, CD80/86/40 low	PD-L1, IL-T3, mTNF	Low IL-12	Yes	Yes	Strongly polarize Th2 response	Chamorro et al. (2009)
Rapamycin	Monocytes	HLA-DR and CD86 like moDCs	Not expressed	Low cytokine production	No	No	Reduced IFNg in stimulated cells	Naranjo-Gomez et al. (2011)
Aspirine	Monocytes	Reduced CD80, CD86, CD40	ILT-3	Not reported	Yes	Yes	Induce *de novo* Tregs^#^	Buckland and Lombardi (2009)
Butyric acid	immDCs	CD80 low; HLA-DR and CD86 like moDCs	Not tested	Low IL-12, high IL-10	Yes	Not tested	Strong phagocytic capacity^#^	Downing et al. (2012)
Dexamethasone	Culture day 3	HLA-DR low, CD80/86/40 low	Low PD-L1, ILT-3	Low IL-12, high IL-10	Strong	Yes	Tregs suppress through soluble factors	Unger et al. (2009)
Vitamin D3	Monocytes	HLA-DR intermediate, CD80/86/40 low	PD-L1, IL-T3, mTNF	Low IL-12, high IL-10	Strong	Yes	Migrate to inflammation, confer infectious tolerance	Kleijwegt et al. (2010, 2011)
Aspergillus proteases	mm.DC	HLA-DR not tested,l CD86 low	ILT-4, RALDH-2, NOS, IDO	IL-8, no IL-10, no IL-12	At a high DC:T ratio	No	Induce anergic T cells^#^	Zimmer et al. (2012)
Semen	Monocytes	HLA-DR low, CD80/86/40 low	Not tested	Low IL-12, TNF, IL-6, high IL-10, TGFb	Not present	TGFb-producing	Maturation-resistant phenotype^#^	Remes et al. (2012)
Trophoblast cells	immDC	HLA-DR, CD86 and CD40 high	Not tested	low IL-12, TNF, high IL-10	Yes	Increase FoxP3 + CD25 +	Migration toward trophoblast cells	Salamone et al. (2012)
Oligonucleotides	Monocytes	Inhibited CD80 and CD86	Unknown	Unknown	Unknown	Unknown	Increase IL-10 producing B cells	Giannoukakis et al. (2011)
Wnt5a	Monocytes	HLA-DR, CD80, CD86, CD40 low	PD-L1, PD-L2 similar to moDC	Low IL-12, high IL-8, IL-10	Yes	IL-10 producing	Wnt5a prevents normal GM-CSF/IL-4 signaling	Valencia et al. (2011)

*inhibition of proliferation of allogeneic T cells.

*^#^characteristics also demonstrated for VD3-DCs*.

## Vitamin D and Dexamethasone Modulated DCs

The active form of the natural immunomodulator Vitamin D (i.e., 1,25(OH)_2_D3), alters the behavior of immune cells shifting T lymphocytes into Tregs and modulating differentiation of human peripheral blood monocytes (Piemonti et al., 2000; Griffin et al., 2001; Adorini et al., 2003; Unger et al., 2009; Baeke et al., 2011), or mouse bone marrow precursors (Griffin et al., 2001). Interestingly, pDCs do not respond to the modulation by 1,25(OH)_2_D3 (Penna et al., 2007). Monocyte-derived DCs generated in the presence of 1,25(OH)_2_D3 (VD3-DC) show a semi-mature phenotype with low MHC-class II expression, low activating co-stimulatory molecules, and production of interleukin IL-10 instead of IL-12 in the culture. In addition, VD3-DCs prevent priming of naïve CD4, or CD8 T cells, induce apoptosis of effector T cells and induce both allo- and auto antigen-specific Tregs from naïve CD4 T cells (van Halteren et al., 2004; Unger et al., 2009; Kleijwegt et al., 2010, 2011, 2013). Similar potential to modulate monocyte development into DCs with tolerogenic phenotype has been shown for dexamethasone (Dex) (Matasic et al., 1999; Piemonti et al., 1999; Rea et al., 2000; Chamorro et al., 2009; Unger et al., 2009). Like VD3-DCs, Dex-DCs possess durable immaturity and a sustained high IL-10 versus low IL-12 production, compared to non-treated monocyte-derived DCs. In mice, application of Dex-DC prior to allograft transplantation significantly prolonged the survival of the grafts in an antigen-dependent fashion. Similar to VD3-DCs, priming with Dex-DC leads to a decrease of IFN-γ-producing cells, while increasing the number of IL-10 producing cells, which could point to the induction of Tr1-like Tregs. However, Tregs induced using Dex-DCs suppress in a bystander fashion independent of antigen (Unger et al., 2009). 1,25(OH)_2_D3 and Dex used in combination generate a third entity of tolerogenic DCs (Combi-DCs), which share most features with VD3-DCs in terms of phenotype and function. Combined treatment with 1,25(OH)_2_D3 and Dex enhanced the modulation of DCs, compared to either compound alone with respect to surface marker expression, inhibition of pro-inflammatory cytokine production, and attenuation of T cell stimulatory capacity (Ferreira et al., 2012). Most importantly, Combi-DCs retain the capacity instilled by 1,25(OH)_2_D3 to induce antigen-specific Tregs. *In vivo*, combined-treated DCs are more potent than IL-10-treated cells to suppress colitis in a murine model (Pedersen et al., 2009).

To further investigate the effects of single vs. combined treatment with 1,25(OH)_2_D3 or Dex as DC modulators, we performed a 2D-DIGE analysis of protein profiles (Ferreira et al., 2012). At the protein level, 1,25(OH)_2_D3 induced major changes in proteins involved in iron metabolism, tricarboxylic cycle, and the purine/pyrimidine metabolism/pentose phosphate pathway. Dex alone changed proteins involved in the response to stress in addition to several proteins included in the proteolysis and the MHCII antigen presentation pathway. Combining both compounds resulted in a unique protein profile of Combi-DCs, although with a major impact of the 1,25(OH)_2_D3. Combi-DCs had a profile closer related to VD3-DCs than to Dex-DCs, which could be due to the experimental setup, since 1,25(OH)_2_D3 treatment started at the beginning of DC differentiation while Dex was added from day 3. Addition of Dex did not seem to interfere with the effects of 1,25(OH)_2_D3 on protein expression in DCs. The protein interaction networks and pathway analysis demonstrated that combined treatment induces drastic changes in metabolic pathways, which might affect the production of or the response to ROS generation resulting in a Combi-DCs that are less sensitive to death by nutrient starvation and have a robust antioxidative machinery possibly ensuring the survival of Combi-DCs at the inflammation site in autoimmune diseases. Since genetic polymorphisms may influence the response of immune cells to 1,25(OH)_2_D3 or Dex alone, these findings ensured us that combining the two immunomodulators to overcome potentially insufficient modulation by a single agent is the way to go with respect to clinical translation for immunomodulatory DC therapy.

## Morphology and Surface Phenotype of VD3-Treated DCs: Activating vs. Inhibitory Co-Stimulatory Molecules

Morphological features of 1,25(OH)_2_D3 and/or Dex modulated DCs are different from non-treated monocyte-derived – moDCs. Non-modulated moDCs form large clusters of non-adherent cells with small dendrites, Dex-DCs are largely non-adherent but show fewer small dendrites and form smaller clusters than moDCs. In contrast, VD3-DCs and Combi-mDCs are adherent with large spindle-shaped dendrites. Interestingly, this spindle-shaped morphology was prominent when VD3- and Combi-DCs were matured with LPS or CD40-L and less evident when DCs were matured using the inflammatory cytokine cocktail (unpublished observation). DCs treated with 1,25(OH)_2_D3 and/or Dex maintain CD14 expression and do not express CD1a, although they show high CD11c and DC-SIGN expression confirming that they are DCs with a semi-mature phenotype and not macrophages or arrested at monocyte stage (Unger et al., 2009; Ferreira et al., 2012). Upon maturation, Combi-mDCs maintain high CD14 expression and high phagocytic capacity compared to moDCs, possibly indicating a resistance to maturation. The functional consequences of residual CD14 expression and phagocytosis remain to be investigated.

Modulated DCs show lower expression of MHC-class II and co-stimulatory molecules CD80 and CD86 when compared to moDCs, upon maturation. VD3-DCs express higher levels of PD-L1 and CD86 compared to Dex-DCs and Combi-DCs. However, VD3-DCs and Combi-DCs show a similar ratio of PD-L1/CD86, being higher than that of Dex-DCs. CD86 and PD-L1 belong to the B7 family of receptors, which interact with the members of the CD28 family of molecules (CD28, CTLA-4, ICOS, and PD-1), generating potent activating or inhibitory signals in T lymphocytes. CD28/B7 interactions mediate co-stimulation and significantly enhance peripheral T-cell responses while CTLA-4 activation decreases T lymphocyte activity and limits the immune response. Similarly, PD-1 receptor interactions with its ligands PD-L1 and PD-L2 on DCs down-regulate T cell immune responses. Despite these similarities, the regulatory roles of the CTLA-4 and PD-1 pathways are different, which may be due to the differential temporal and spatial expression patterns of their ligands. CTLA-4 signaling seems to be required early in the lymph node during initiation of an immune response, while PD-1 pathway acts late in the periphery to limit T-cell activity locally (Greenwald et al., 2005). PD-L1 on VD3-DC is crucial for the acquisition of Treg function by CD4+ T cells and blockade of PD-L1 on VD3-DC during T cell priming generated Th1-like T cells incapable of suppressing allo-reactive T-cell proliferation and producing IL-10 (Unger et al., 2009). These observations suggest that PD-L1/PD-1 interactions impair the generation of effector T cells in favor of Treg formation. The underlying mechanism may involve reverse signaling by PD-L1 into DC leading to decreased expression of the positive co-stimulatory molecules CD80, CD86, and CD40 and increased IL-10 production.

Inducible T cell costimulator (ICOS) has overlapping functions with CD28 in early T-cell activation, and has emerged as an important receptor in the immune system to regulate T-cell effector functions (de la Fuente et al., 2012). The ICOS/ICOSL pathway is also involved in immune tolerance since ICOS regulates the survival of both effector memory T cells and FoxP3+ Tregs during homeostasis or antigen-specific immune response (Burmeister et al., 2008). Modulated VD3- and Combi-DCs express ICOSL at similar levels as the non-modulated moDC. The contribution of ICOSL in the “downstream” tolerogenic mechanisms installed by VD3-DCs will be discussed later in this review.

## Anti-inflammatory Cytokine Production and Relation to “Tolerogenic” Phenotype

A shift in cytokine production to predominant IL-10 and reduced IL-12 production by 1,25(OH)_2_D3-treated DCs has been observed *in vitro* in multiple studies with human as well as murine DCs (Black et al., 1997; Ferreira et al., 2011). The turn-off signal for the IL-12 and related cytokines could be IL-10, which blocks IL-12 synthesis, down-regulates the expression of co-stimulatory molecules and potentiates tolerogenicity (Steinbrink et al., 1997; Rutella et al., 2006). Proteomics analysis shows that 1,25(OH)_2_D3 and Dex induce changes in DCs other than IL-10 production alone. Yet, the contribution of IL-10 to the tolerogenic phenotype might be larger than anticipated. TLR-activation induces a metabolic transition in DCs from oxidative phosphorylation to aerobic glycolysis and IL-10 blocks this transition. By switching toward oxidative phosphorylation, DCs are less reliant on glucose for survival and function, and thus less sensitive to death by nutrient starvation (Steinbrink et al., 1997; Rutella et al., 2006; Ferreira et al., 2009). IL-10 is also important for the immunomodulatory effects of VD3-DCs, attenuating Th1 driving forces and being protective in settings of autoimmunity. Together with TGF-β, IL-10 expression is a hallmark of the presence of regulatory T cells. However, IL-10 does not seem to be involved in the actual suppression by Tregs.

Tumor necrosis factor (TNF) is a pleiotropic cytokine most known for pro-inflammatory (Th1) functions in multiple autoimmune diseases (Braun et al., 2002; Feldmann and Maini, 2003). However, a beneficial effect of TNF in autoimmunity is also acknowledged (Robinson et al., 2001; Ramos-Casals et al., 2008; Ko et al., 2009; Tack et al., 2009). This dual role of TNF is seen in other pathologic conditions such as infectious diseases and cancer (Gimenez, 2003; Calzascia et al., 2007). Diverse roles of TNF in the immune response may be partly inferred by two forms of TNF: a membrane-bound TNF (mTNF), which is cleaved from the membrane and released as a soluble cytokine (sTNF) (Black et al., 1997). We demonstrated that VD3-DC produce increased amounts of sTNF upon LPS stimulation. Yet, the expression of mTNF distinguishes VD3-DCs from moDCs (Black et al., 1997; Kleijwegt et al., 2010). We further demonstrated that mTNF is essential for the induction of allo-specific suppressive T cells by VD3-DCs through mTNF-TNFRII interaction, adding the role of mTNF in the induction of immune tolerance to the list of multiple functions of this cytokine.

Indoleamine 2,3-dioxygenase (IDO) and aryl hydrocarbon receptor (AhR) have been implicated as negative regulators of the inflammatory response by modulating the Th1/Th2 balance (Negishi et al., 2005), and regulation of Treg and Th17 cell differentiation (Quintana et al., 2008). We did not find evidence for the contribution of these molecules in modulated DCs.

## PRRs and Resistance to Maturation

Several synthetic TLR ligands activate DC subsets and promote their adjuvant pro-inflammatory capacity, which can be a useful tool in certain types of vaccines. DC express a broad repertoire of TLRs with 10 members of the family described to date in human (Medzhitov, 2001). TLRs trigger the signals that provide DC maturation and the initiation of adaptive immune responses against pathogens. The most known ligand for TLR4 is LPS (Medzhitov, 2001), whereas TLR2 discriminates lipoproteins from Gram-positive bacteria in association with TLR1 or TLR6 (Schwandner et al., 1999; Takeuchi et al., 2002). In addition, self-proteins can be recognized by both TLR2 and TLR4 (Asea et al., 2002).

The TLR repertoire on tolerogenic DCs modulated *in vitro* with IL-10, dexamethasone, or Vitamin D appears similar to that of immunogenic DCs, although with slight differences in the expression levels of TLR2 and TLR4 (Chamorro et al., 2009). However, tolerogenic DCs responded differently to TLR mediated signals than immunogenic DCs showing partial maturation with low to intermediate levels of CD80, CD86, and CCR7, reduced ability to stimulate allogeneic T cells and secretion of the suppressive cytokine IL-10 combined with very low production of IL-12 or the related cytokines IL-23 and IL-27. The finding that partially matured DCs respond to TLR2 stimulation with an anti-inflammatory program could shed light on why patients with severe sepsis undergo long-term systemic and local immunosuppression despite their increased TLR2 expression (Armstrong et al., 2004), and how TLR2-derived signals from *Candida albicans* or *Schistosome* infections drive immunosuppression by IL-10 production and Treg induction (van der Kleij et al., 2002; Netea et al., 2004). Indeed, TLR2 participates in the induction of peripheral tolerance (Dillon et al., 2006), in the promotion of T regulatory responses leading to protection against autoimmune diseases *in vivo* (Manicassamy et al., 2009). Thus, the TLR2 up-regulation and activation on VD3- and Dex-treated DCs might enhance their tolerogenic properties.

*In vivo* application of DCs in patients faces possible pro-inflammatory triggering and the tolerogenic DC vaccine should not divert into pro-inflammatory DC upon injection. Preservation of tolerogenic function in the settings of an activated immune system (resistance to maturation) is therefore considered a prerequisite of tolerogenic potential for immunomodulatory vaccines. Pro-inflammatory DC maturation is induced when DCs sense microbes through PRRs, interact with T cells (through CD40) or get exposed to inflammatory cytokines. We mimicked *in vivo* DC activation by re-stimulating matured tolerogenic DCs with LPS, CD40-L, or the cocktail of cytokines (IL-6, TNF, IL-1β, and PGE2), and demonstrated that DCs modulated with Vitamin D alone or combined with dexamethasone maintained a stable regulatory phenotype, IL-10 production, and low stimulation of allogeneic T cells upon repeated maturation with either LPS, CD40-L, or cytokine mix. Similarly, a study comparing resistance to re-stimulation with LPS of the rapamycin-, dexamethasone-, and Vitamin D-treated DCs, demonstrated a stable high IL-10 and no IL-12/IL-23 production (Naranjo-Gomez et al., 2011).

## Modulated DC are Equipped to Migrate to Inflamed and Secondary Lymphoid Tissues – Chemokine Production upon TLR Triggering

To be effective in silencing reactive T cells or inducing tissue-specific Tregs *in vivo*, it is of key importance that injected modulated DCs reach lymphoid tissues or the inflamed site. We therefore examined the expression of several chemokine receptors. Immature Dex-, VD3-, and moDCs expressed significant levels of CCR4 and CCR5, but no CCR7 (Unger et al., 2009). Upon maturation, CCR4 and CCR5 were down-regulated, whereas CCR7 was up-regulated by all DCs indicating the ability to migrate to secondary lymphoid tissues. CXCR3 was expressed on the surface of the modulated DCs, which may guide these cells to the inflammatory lesion in the pancreas to counteract autoreactive T cells around the distressed islets (van Halteren et al., 2005; Roep et al., 2010).

## Preventing Priming of Naïve T Cells – Neglection or Active Inhibition?

**Figure 2:**
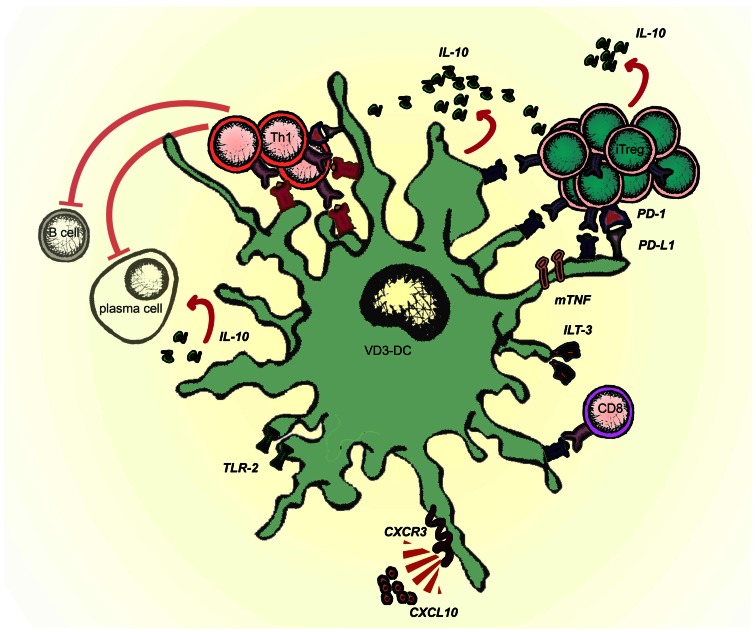
**Molecules involved in the tolerogenic function of Vitamin D-modulated DCs (VD3-DCs)**. VD3-DC express MHC class II in combination with low co-stimulatory molecules (CD80/86) and high expression of PD-L1, which enable antigen-dependent elimination of Th1 effector T cells and induction of antigen-specific adaptive regulatory T cells (iTregs). VD3-DCs express additional regulatory molecules: TLR2, ILT-3, and membrane-bound TNF (mTNF), and produce IL-10, which potentiate the tolerogenic function of VD3-DCs. VD3-DCs also express chemokines, which enable the migration both to lymph nodes (CCR7) and to the site of inflammation (CXCR3).

Schematic representation of molecules involved in the tolerogenic function of VD3-modulated DCs is given in Figure 2. VD3-DC and Combi-DC impair priming of naïve CD4 and CD8 T cells (Unger et al., 2009; Kleijwegt et al., 2013). Naïve T cells require antigen recognition, co-stimulation, and activating cytokines (mostly IL-12) to become effector T cells. VD3-DC and Combi-DCs express lower levels of co-stimulatory molecules and IL-12, providing incomplete stimulation to T cells. However, our data show that besides hampered stimulation, an active repression prevents priming of naïve T cells (Kleijwegt et al., 2013). Combi-DCs produce TNF and TGF-β (Chamorro et al., 2009; Kleijwegt et al., 2010), but neither of these cytokines contributed to the obstructed priming of CD8 T cells. Since Combi-DCs interfere with priming only when they present the cognate antigen to the T cell, mechanisms related to TCR signaling may be responsible for this effect. This could happen through failing trans-presentation of IL-15, IL-2 (Combi-DCs lack CD25 expression), or high ILT-3 expression, as demonstrated in other systems (Chamorro et al., 2009; Frasca et al., 2010; Kleijwegt et al., 2010; Huntington et al., 2011).

## Killing of T Cells by Tolerogenic DCs – The Story of Mr. and Ms. Smith

Tolerogenic mechanisms of VD3-DCs include deletion of naïve T cells since the cell numbers after the co-culture with VD3-DCs are reduced compared to the cultures with non-treated moDCs. The ability of DCs to kill target cells that could serve in anti-tumor immunity or T-cell killing in autoimmunity and transplantation has been described (Chauvin and Josien, 2008). A recent study in pulmonary tuberculosis patients confirms the cytotoxic activity of DCs as a mechanism of negative regulation of T lymphocytes (Sakhno et al., 2012). In case of VD3-DCs, this action is antigen-dependent but not restricted to naïve T cells since VD3-DCs also block IFNγ and IL-10 production and induce active killing of a diabetogenic Th1 clone in an antigen-specific manner (van Halteren et al., 2002). When interacting with memory CD8 T cells, Combi-DCs initially expand this cell pool, which collapses after the subsequent re-stimulation (Kleijwegt et al., 2013). This is caused by an increased cell death in the second co-culture. To test whether memory CD8 T cells undergo increased apoptosis in the second culture due to the primary interaction with tolerogenic DCs, memory CD8 T cells initially stimulated with tolerogenic DCs were subsequently re-stimulated with moDCs. This only partially rescued CD8 T cells from the collapse in the second culture, implying that during the first stimulation tolerogenic DCs endorse memory CD8 T cells with features underlying their subsequent ceased growth. The depletion of CD8 T cells by Combi-DCs could result from a combined action of the withdrawal of positive signals (co-stimulation and cytokines) and inhibition by secreted regulatory factors (such as IL-10 and TGF-β), creating a less favorable environment for cytotoxic T cell survival and expansion (Bots et al., 2007; Toda et al., 2011). This process could also involve expression of CXCR3 facilitating memory degeneration, inhibition of autocrine IL-2 production, or induction of pro-apoptotic signals as described in other models (Kurachi et al., 2011; Takata et al., 2012).

The finding that tolerogenic DCs induce elimination of effector CD8 T cells initially appeared to offer a valuable tool to tackle the destructive forces such as autoreactive CD8 T cells in the pancreas of patients with type 1 diabetes by loading diabetogenic MHC-class I peptides onto tolerogenic DCs. However, a draw-back in this respect was our finding that antigen-loaded tolerogenic DCs were eliminated by the cytotoxic CD8 T cells through a Granzyme B-dependent mechanism (Kleijwegt et al., 2013). DCs have the ability to protect themselves from being killed using protective molecules like Serpin 9 or Cathepsin B (Bots et al., 2007) or by up-regulation of antiapoptotic proteins, such as Bcl-xL (Gutierrez et al., 2007). Indeed, tolerogenic DCs were more protected from killing than moDCs or B cells. Yet, in order to preserve the immunomodulatory action of tolerogenic vaccine on other cells types, loading of MHC-class I epitopes does not appear to add to MHC-class II epitopes on tolerogenic DCs.

## Vitamin D3-Treated DCs Induce Infectious Tolerance through Antigen-Specific Tregs

As established above, VD3- and Combi-DCs induce Tregs that suppress in an antigen-dependent manner, which is specialized feature of 1,25(OH)_2_D3-treated DCs. Rapa-DC-primed T cells exhibit reduced alloproliferation along with a concomitant expansion of naturally occurring tTregs (Kang et al., 2008; Simonetta et al., 2010), and Dex-DCs induce adaptive Tregs that suppress in an antigen-independent fashion (Unger et al., 2009). PD-L1 and mTNF play important roles in the mechanisms by which VD3-DCs induce allo-reactive or proinsulin specific Tregs (Kleijwegt et al., 2011).

Induced (adaptive) proinsulin-peptide specific Tregs (iTregs) suppress diabetogenic Th1 cells via linked suppression and do not need IL-10 or TGF-β to perform their suppressive functions (Kleijwegt et al., 2011). Tregs may exert their antigen-specific function via cytolysis of the APCs, metabolic disruption, or by modulating the immunogenic function of DCs (Vignali et al., 2008). We have demonstrated that iTregs induced by VD3-DC perform two of three described functions and the contribution of iTregs to metabolic disruption remains to be investigated. iTregs re-educate pro-inflammatory moDCs in an antigen-specific manner by changing the phenotype (@DC), which differs from VD3-DCs used to generate iTregs: VD3-DCs have low CD80 and CD86, high ILT-3 and PD-L1, low ICOSL, and no B7-H3 expression (Unger et al., 2009), whereas @DCs retain high expression of HLA-DR and co-stimulatory molecules CD80 and CD86, and low expression of inhibitory molecules PD-L1, ILT-3, and ILT-4 (Kleijwegt et al., 2011). The difference in phenotype may originate in the mode of modulation: VD3-DCs result from modulation of monocytes with vitamin D3 during differentiation, whereas iTregs alter fully differentiated mature moDCs into @DC. The specific alteration of antigen-bearing moDCs, which persisted upon removal of iTregs, was characterized by up-regulation of B7-H3 and ICOSL. Both molecules are involved in immune regulation: B7-H3 preferentially dampens Th1-mediated responses (Suh et al., 2003), whereas ICOSL promotes IL-10 secretion by T cells (Witsch et al., 2002) and induces Tr1 cells that produce IL-10 and inhibit experimental airway inflammation in mice (Akbari et al., 2002; Conrad et al., 2012). The induction of IL-10–producing T cells by human @DCs was indeed mediated through ICOSL.

**Figure 3:**
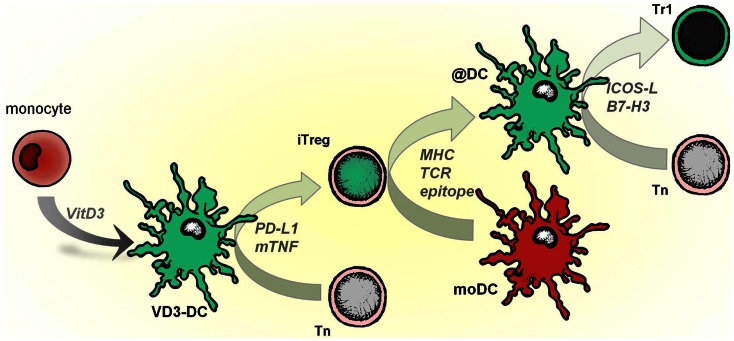
**VD3-DCs confer infectious tolerance through antigen-specific adaptive regulatory T cells (iTregs)**. Vitamin D modulates monocytes to differentiate into tolerogenic DCs (VD3-DCs), which induce iTregs. This process is mediated by PD-L1 and membrane-bound TNF (mTNF). iTregs, in turn, functionally modify pro-inflammatory moDCs to become anti-inflammatory DCs (@DCs), which present the cognate antigen of iTregs. As a consequence, @DCs upregulate inhibitory receptors ICOSL and B7-H3, lose the capacity to stimulate Th1 responses and instead induce IL-10–producing T cells from the naive T cell pool.

Infectious tolerance is a process in which a tolerance-inducing state is transferred from one cell population to another and is supported by different mechanisms, including IL-35, TGF-β production, or essential amino acid-depleting enzymes (Jonuleit et al., 2002; Cobbold et al., 2009; Collison et al., 2010). VD3-DCs transfer tolerogenic properties to mature pro-inflammatory DC via iTregs, which operate in an antigen-dependent manner (modeled in Figure 3). When applied as an intervention therapy, this implies that Tregs specific for a single auto antigen generated by tolerogenic DCs could alter other local DCs that subsequently reduce autoimmune responses to a diverse range of tissue-associated antigens.

Interplay between DCs and Tregs is essential for the immune tolerance since a direct correlation between DC numbers and Treg cells has been found as part of a feedback-control mechanism (Darrasse-Jeze et al., 2009). DCs promote and enhance the suppression by Treg cells and in turn, Tregs condition other DCs to upregulate inhibitory molecules and modulate effector responses. Instillation of VD3-DCs in this loop would support the regulatory and anti-inflammatory forces in the setting of autoimmune response that has escaped the regular control.

## Concluding Remarks – Toward a Tolerogenic Immunotherapy with DCs

Getting hold of a method to obtain efficient tolerogenic DCs in the clinical setting is a holy grail that will enable reshaping the immune response to particular needs. This may be accomplished using *ex vivo* modulated DCs. These cells might not be the same as tolerogenic DCs that exist *in vivo* and might need to be repetitively injected to achieve a continuous tolerogenic treatment. However, the *ex vivo* generation of tolerogenic DCs permits the propagation of sufficient amounts of cells in controlled conditions and loading with an antigen of choice thereby enabling targeted tolerizing immunotherapies.

Tolerogenic DCs modulated using the biologically active form of vitamin D (1,25(OH)_2_D3) are a promising tool for tolerance induction in clinic. Years of investigation of these tolerogenic DCs have helped understand the contribution of different PRRs and secondary signals in facilitating tolerogenic DCs to prime antigen- and tissue-specific Tregs by mechanisms such as linked suppression and infectious tolerance. These learned lessons helped understanding how a cross-talk between DCs and T cells translates into immune regulation and may support finding therapies with small molecules that recapitulate the mode of action of cell therapy.

## Conflict of Interest Statement

The authors declare that the research was conducted in the absence of any commercial or financial relationships that could be construed as a potential conflict of interest.
